# Reversed Halo Sign: Presents in Different Pulmonary Diseases

**DOI:** 10.1371/journal.pone.0128153

**Published:** 2015-06-17

**Authors:** Xi Zhan, Lei Zhang, Zheng Wang, Mulan Jin, Min Liu, Zhaohui Tong

**Affiliations:** 1 Department of Respiratory and Critical Care Medicine, Beijing Chao-Yang Hospital, Capital Medical University, Beijing, 100020, China; 2 Beijing Key Laboratory of Respiratory and Pulmonary Circulation, Beijing Institute of Respiratory Medicine, Beijing, 100020, China; 3 Department of Radiology, Beijing Chao-Yang Hospital, Capital Medical University, Beijing, 100020, China; 4 Department of Pathology, Beijing Chao-Yang Hospital, Capital Medical University, Beijing, 100020, China; University of Alabama at Birmingham, UNITED STATES

## Abstract

**Objective:**

To observe the incidence of reversed halo sign in different pulmonary diseases and the pathological correspondence of reversed halo sign.

**Methods:**

Retrospectively studied the high resolution computer tomography scans of all the patients who were admitted in our department with abnormal pulmonary imaging, from 1st of January 2011 to 31st of December 2013, and all the cases with reversed halo sign on the high resolution computer tomography were collected. Clinical data such as pathological findings and confirmed diagnosis of the patients with reversed halo sign on the high resolution computer tomography scan were collected and summarized.

**Results:**

Of 1546 abnormal High resolution computer tomography scans 108 had a reverse halo sign present, including 108 cases were observed with reversed halo sign in the high resolution computer tomography, including 40 cases of pulmonary tuberculosis, 43 cases of cryptogenic organizing pneumonia, 16 cases of lung cancer, 7 cases of sarcoidosis, and 1 case of pulmonary cryptococcosis, 1 case of granulomatosis with polyangiitis. Reversed halo sign had a higher incidence in granulomatous diseases (16.28%) compared with non-granulomatous diseases (9.97%).

**Conclusions:**

Reversed halo sign is relatively non specific; it can be observed in different lung diseases, and different phases of diseases; reversed halo sign is more commonly found in granulomatous diseases compared with non-granulomatous diseases, and is most commonly observed in pulmonary tuberculosis among the granulomatous diseases, and in cryptogenic organizing pneumonia among the non-granulomatous diseases.

## Background

Reversed halo sign was first reported in 1996 in 2 cases of cryptogenic organizing pneumonia (COP) by Voloudaki et al, and described as central opacity surrounded by a ring-shaped consolidation on computer tomography (CT) scan [1]. In 2003 the special sign was named as “reversed halo sign “by Kim et al [2], who compared the incidence of reversed halo sign on high resolution computer tomography (HRCT) of 31 cases of cryptogenic organizing pneumonia(COP) with that of 30 cases of non-COP diseases, and considered the reversed halo sign to be a specific sign of COP. However, later reversed halo sign was also reported to be shown in other diseases, such as pulmonary paracoccidioidomycosis (in 2005) [3], pulmonary lymphomatoid granulomatosis (in 2007) [4], Wegener’s granulomatosis (in 2007) [5] and pulmonary tuberculosis [6]. In 2008 the term “reversed halo sign” was recorded in the glossary of terms of the Fleischner Society [7], and was defined as a focal rounded area of ground-glass opacity surrounded by a more or less complete ring of consolidation [7]. From then on, other diseases such as sarcoidosis[8], invasive pulmonary fungal infections[9], were also reported to have reversed halo sign on the high resolution computer tomography(HRCT), and Reversed halo sign has most frequently been observed in pulmonary tuberculosis and cryptogenic organizing pneumonia(COP)[10,11]. However, most of the papers were case reports, and the reversed halo sign in different pulmonary diseases based on the pathological correspondence has never been systemically studied before. This research aimed to observe the incidence of reversed halo sign in different pulmonary diseases and its pathological correspondence, by retrospectively studying the HRCT of all the patients admitted in the Department of Respiratory and Critical Care Medicine of Beijing Chaoyang Hospital with abnormal pulmonary imaging, from 1st of Jan 2011 to 31st of December 2013, including 1546 patients.

## Materials and Methods

Beijing Chaoyang hospital is the largest tertiary hospital in east Beijing, and the Department of Respiratory and Critical Care Medicine contains 198 beds. Between 1st of January 2011 and 31st of December 2013, all the patients who were admitted in our department with abnormal lung imaging were retrospectively studied. All their HRCT scans were re-read, and those with the reversed halo on the HRCT were recruited for this study. Other clinical data including age, gender, confirmed diagnosis and pathological findings were collected too. Reversed halo sign was defined as a focal rounded area of ground-glass opacity surrounded by a more or less complete ring of consolidation [7].

### High-Resolution CT

The high resolution computer tomography(HRCT) were performed on a 64- slice CT scanner (GE LightSpeed VCT USA) with the following parameters: technical parameters for HRCT: 0.625mm collimation and 3mm interval using a high-spatial-frequency reconstruction algorithm. Reconstructed slice thickness = 3.75mm, Rotation time = 0.8sec, pitch = 0.969:1, KV = 100-120KV, mA = 200-320mA, but modulated according to the personal BMI. Images were acquired at mediastinal (width, 400 HU; level, 40 HU) and parenchymal (width, 1500 HU; level, −700 HU) window settings. The time interval between HRCT and biopsy was 3 days. The biopsy system was MANAN SUPER-CORE Biopsy Instrument (MD TECHNOLOGIES, USA), 18G/9cm. No contrast media was used.

Two chest radiologists with more than 10 years of experience independently read the CT images scans, and decisions concerning the findings were reached by consensus.

### Statistics

A Pearson’s χ2 test was used to test for difference. The data was processed with SPSS software version 16.0.

The authors had approached an ethics committee prior to the beginning of the study, as this was a retrospective study on existing data it did not require an ethics statement. No authors had interaction with the patients. Written informed consent for the re-use of the data was obtained from the patients. All the patients’ information was de-identified prior to analysis.

## Results

During the period between 1st of January 2011 and 31st of December 2013, the number of patients who were admitted in our department with abnormal lung imaging on high resolution computer tomography(HRCT) was 1546, and 108 of them were found to have the reversed halo sign on HRCT with different diagnosis, including cryptogenic organizing pneumonia(COP), pulmonary tuberculosis, lung cancer, sarcoidosis, pulmonary cryptococcosis, granulomatosis with polyangiitis(GPA), which were divided into 2 groups based on their differing pathology: granulomatous diseases and non-granulomatous diseases. In the group of granulomatous diseases (Table 1), 40 were pulmonary tuberculosis(out of all the 138 cases of pulmonary tuberculosis diagnosed in the same period, 28.99%), 7 were sarcoidosis (out of all the 133 cases of sarcoidosis diagnosed in the same period, 5.26%), 1 was pulmonary cryptococcosis (out of all the 13 cases of invasive pulmonary fungal infection with pathological diagnosis who were diagnosed in the same period, 7.69%), 1 was granulomatosis with polyangiitis(GPA) (out of all the 17 cases of GPA diagnosed in the same period, 5.88%); In the group of non-granulomatous diseases (Table 2), 43 were cryptogenic organizing pneumonia (COP) (out of all the 179 cases of COP diagnosed in the same period, 24.02%), 16 were lung cancer (out of all the 413 cases of lung cancer diagnosed in the same period, 3.87%). The Pearson’s χ2 test was used to test for difference between the two groups. The incidence of reversed halo sign was significantly higher in the group of granulomatous diseases(16.28%), than that of the group of non-granulomatosis diseases(9.97%), P = 0.006. Reversed halo sign was most commonly observed in pulmonary tuberculosis (28.99%) among the granulomatous diseases and in COP (24.02%) among the non-granulomatous diseases. The data was processed with SPSS software version 16.0.

**Table 1:** Granulomatous diseases.

	Reversed halo sign	non-Reversed halo sign	Total
Tuberculosis	40(28.99%)	98(71.01%)	138
Sarcoidosis	7(5.26%)	126(94.74%)	133
GPA	1(5.88%)	16(94.12%)	17
Cryptococcosis	1(7.69%)	12(92.31%)	13
Total	49(16.28%)	252(83.72%)	301

**Table 2:** Non-granulomatous diseases.

	Reversed halo sign	non-Reversed halo sign	Total
COP	43(24.02%)	136(75.98%)	179
Lung cancer	16(3.87%)	397(96.13%)	413
Total	59(9.97%)	533(90.03%)	592

**Fig 1:**
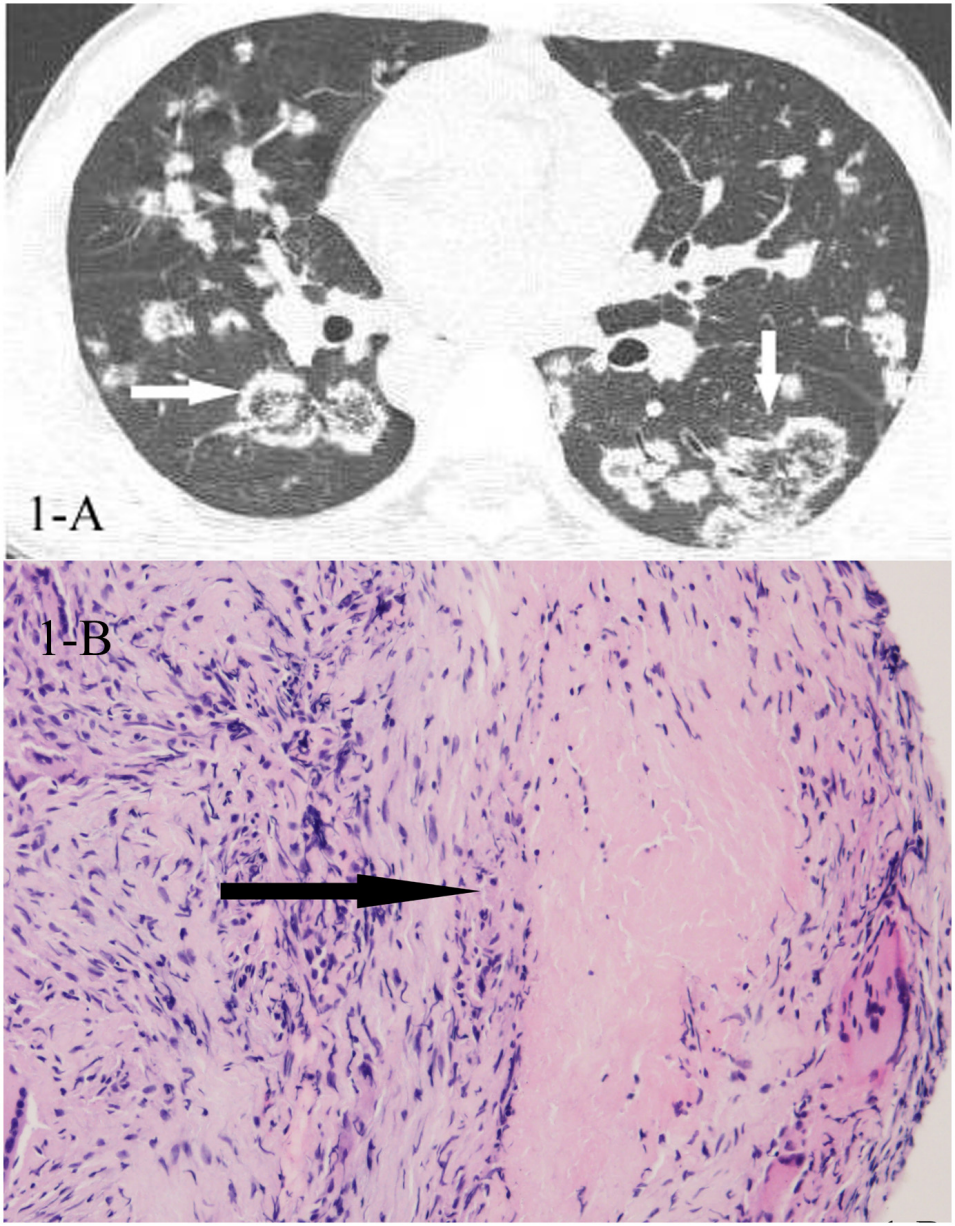
A,B. A 26-year-old male diagnosed with tuberculosis pathologically. HRCT showed reversed halo sign(1-A,white arrow), and the CT-guided lung biopsy of the “ring” showed granulomas with caseating necrosis(1-B,black arrow, HE stain, 400x magnification).

**Fig 2:**
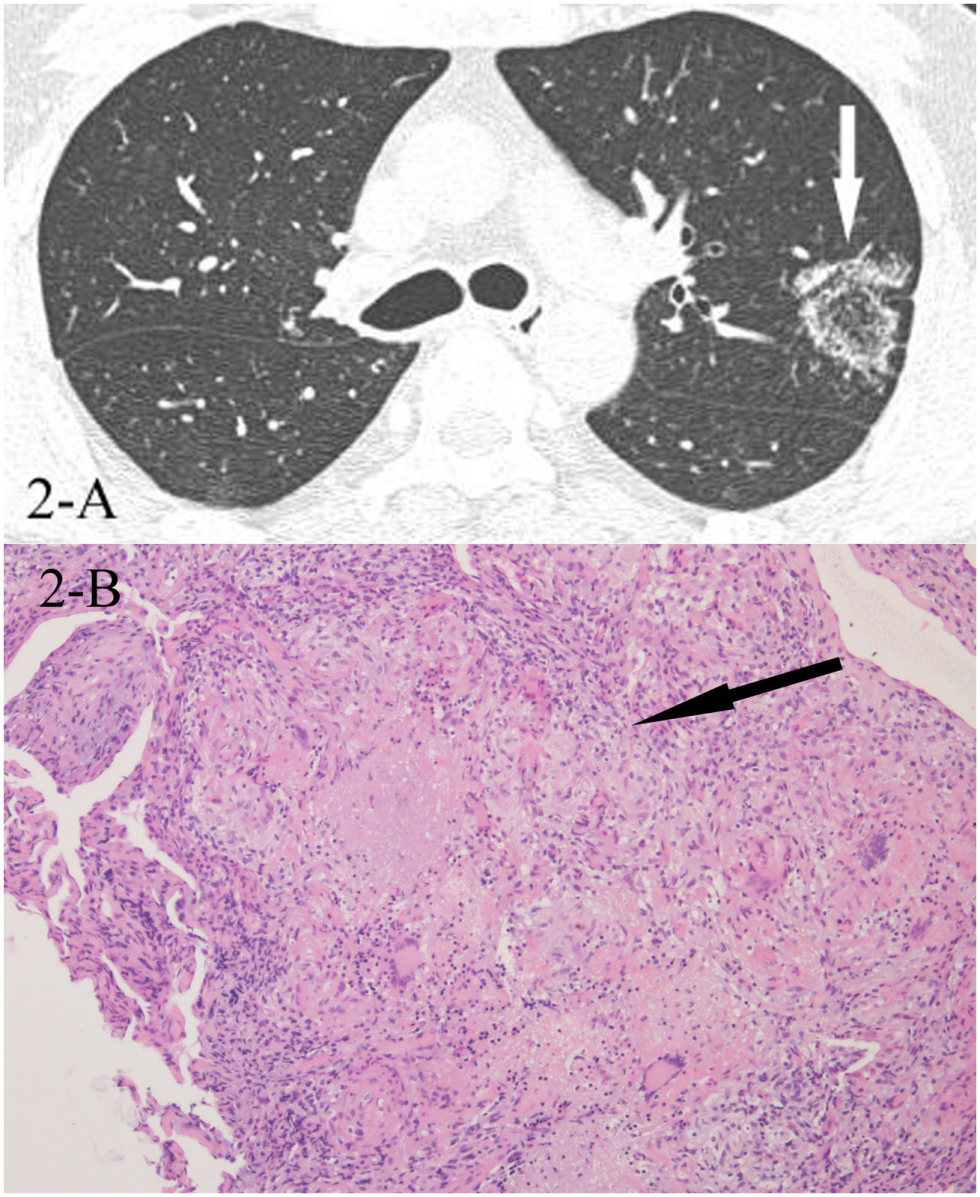
A, B. A 37-year-old male diagnosed with tuberculosis pathologically. HRCT showed reversed halo sign(2-A,white arrow), and the CT-guided lung biopsy of the “ring” showed granulomas with caseating necrosis(2-B,black arrow, HE stain, 100x magnification).

**Fig 3:**
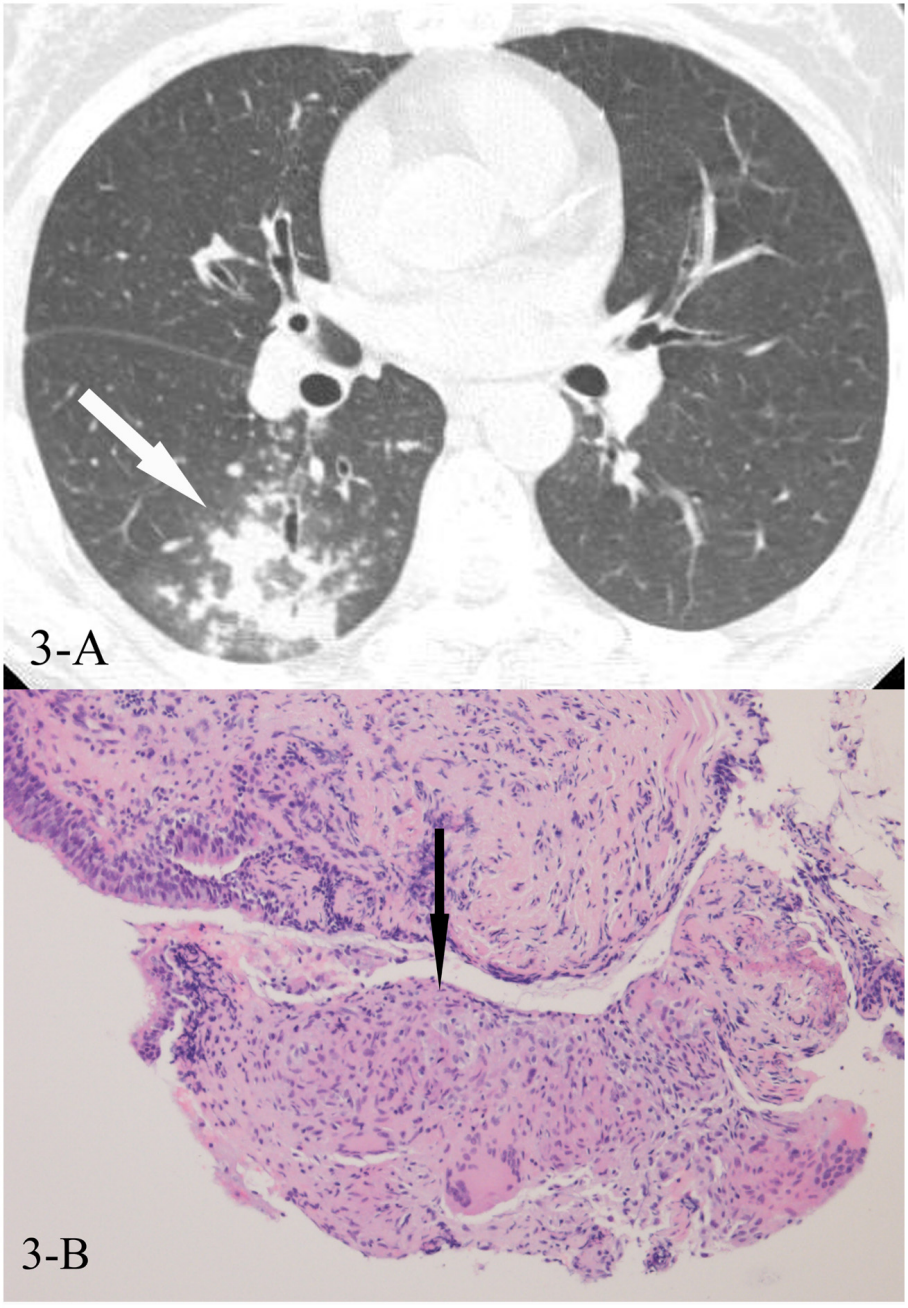
A, B. A 52-year-old female diagnosed with tuberculosis by smear positive sputum(at the third time of sputum smear). HRCT showed reversed halo sign(3-A,white arrow), and the CT-guided lung biopsy of the “ring” showed granulomas (3-B,black arrow, HE stain, 200x magnification).

**Fig 4:**
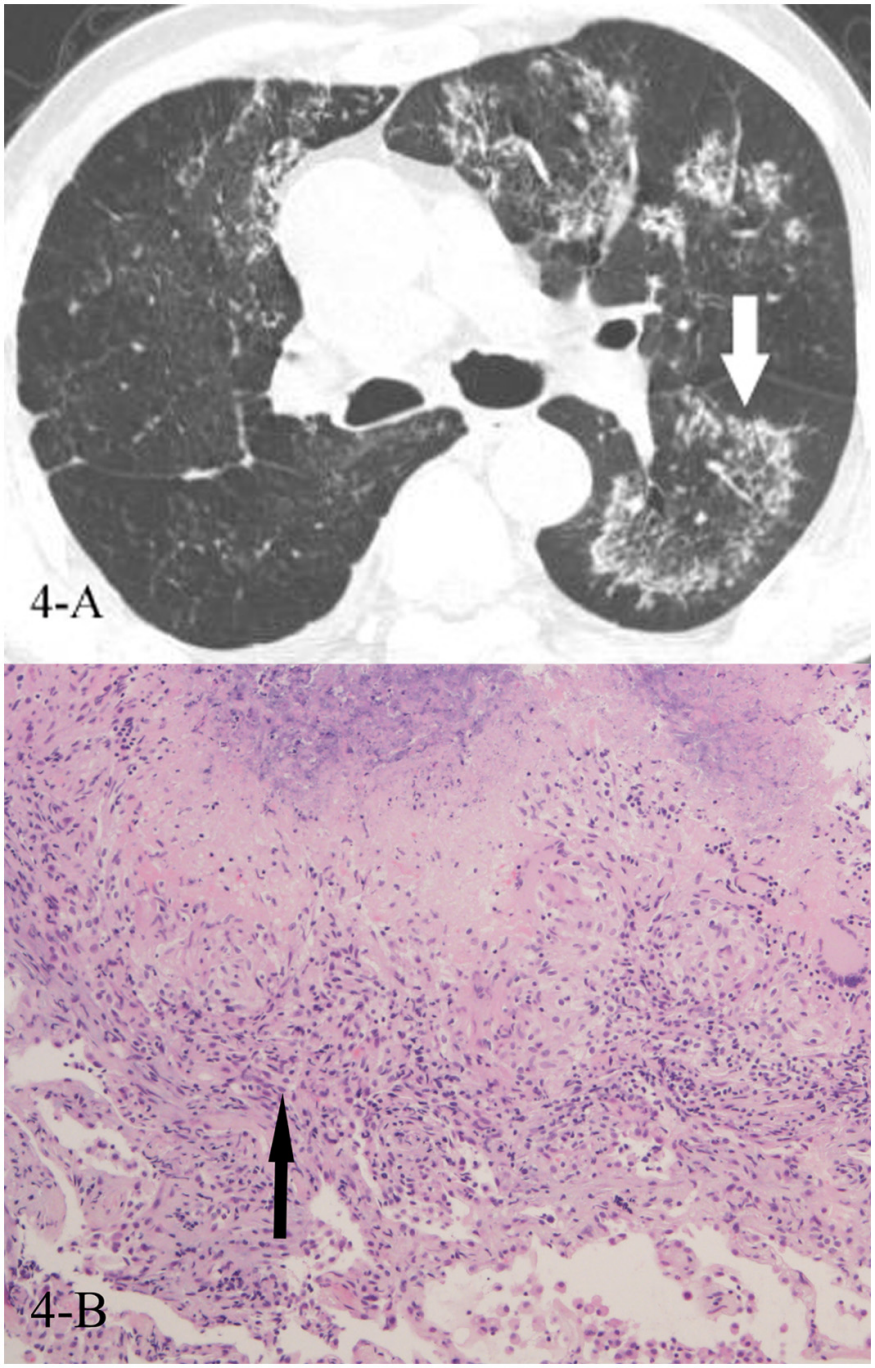
A, B. A 72-year-old male diagnosed with tuberculosis pathologically. The HRCT showed reversed halo sign(4-A,white arrow), and the CT-guided lung biopsy of the “ring” showed granulomas with caseating necrosis(4-B,black arrow, HE stain, 100x magnification).

**Fig 5:**
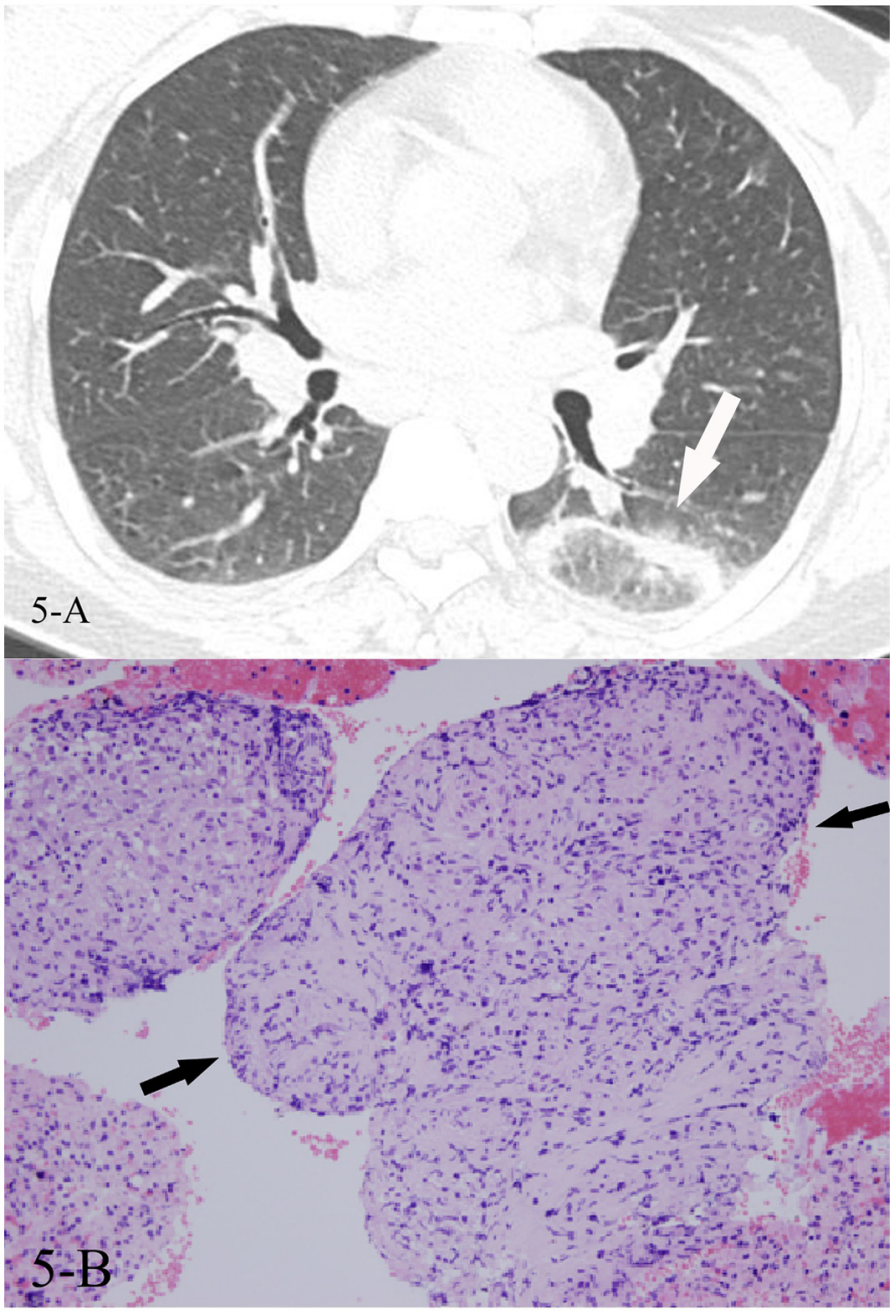
A, B. A 54-year-old female diagnosed with sarcoidosis pathologically. The HRCT showed reversed halo sign(5-A,white arrow) and bilateral hilar lymph nodes enlargement, and the CT-guided lung biopsy of the “ring” showed multiple non-caseating necrotic granulomas(5-B, black arrows, HE stain,400x magnification), which was consistent with the bronchoscopic mucosa biopsy.

**Fig 6:**
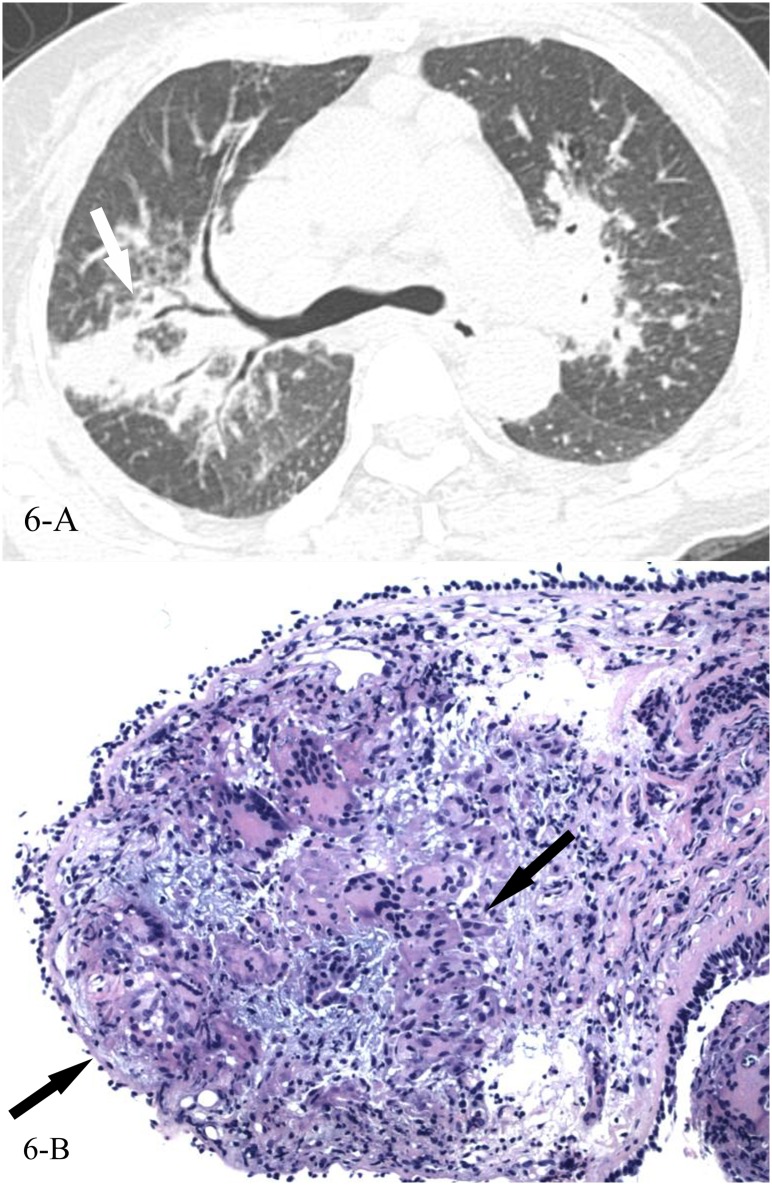
A, B. A 55-year-old female diagnosed with sarcoidosis pathologically. The HRCT showed reversed halo sign(6-A,white arrow) and bilateral hilar lymph nodes enlargement, and the bronchoscopic mucosa biopsy showed multiple non-caseating necrotic granulomas(6-B, black arrows, HE stain,200x magnification).

**Fig 7:**
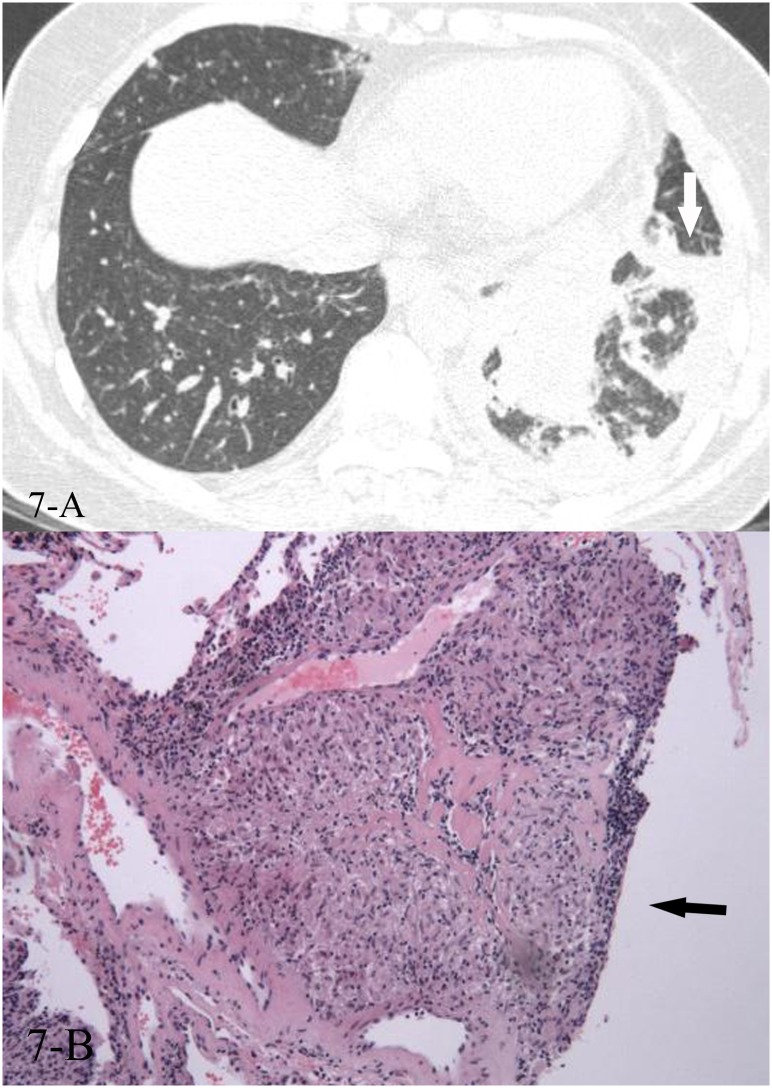
A, B. A 50-year-old female diagnosed with sarcoidosis pathologically. The HRCT showed reversed halo sign(7-A,white arrow), and the CT-guided transthoracic lung biopsy of the focal “ring” showed non-caseating necrotic granulomas(7-B, black arrow, HE stain, 200x magnification).

**Fig 8:**
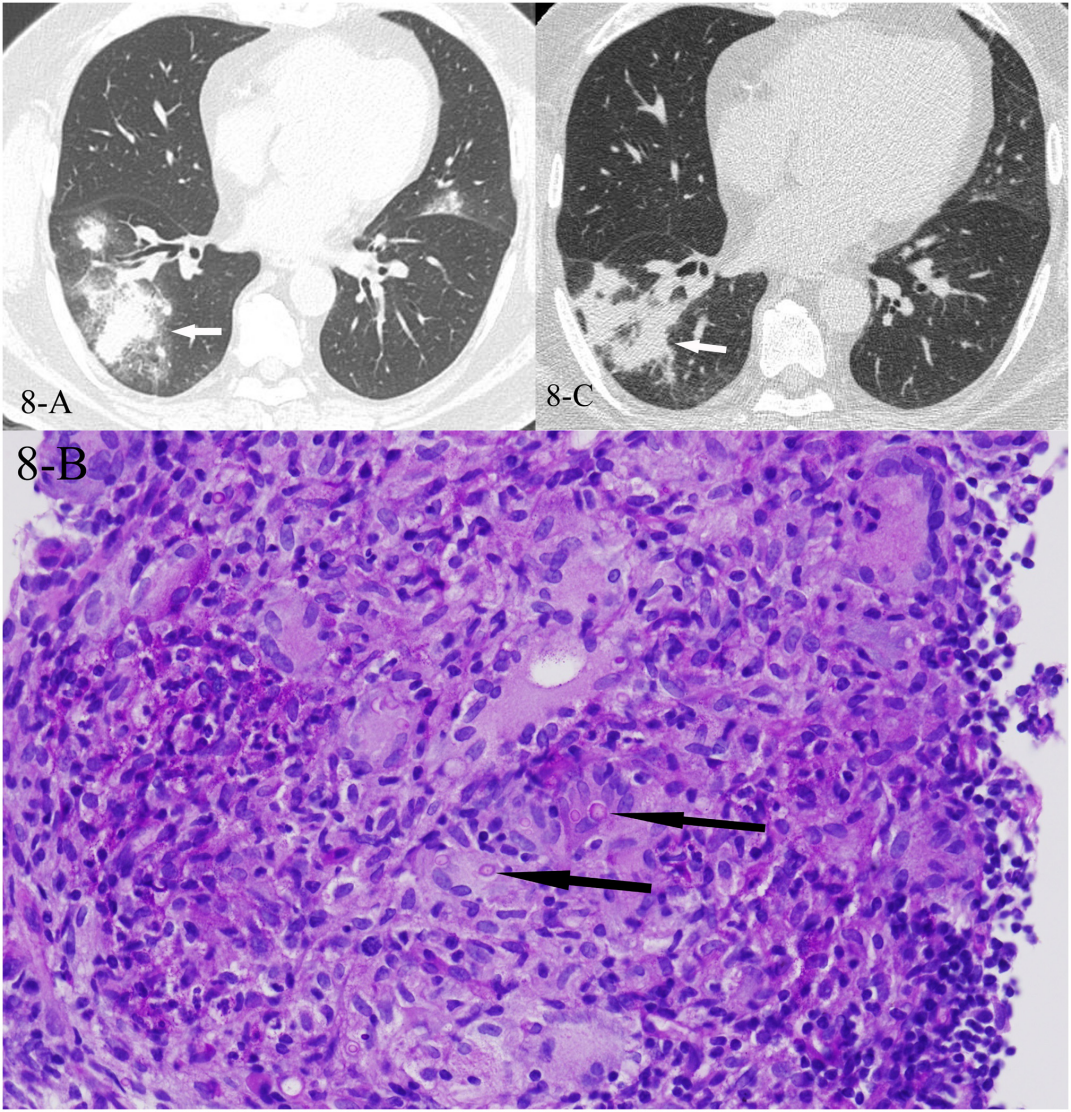
A, B, C. A 55-year-old male diagnosed with pulmonary cryptococcosis pathologically. The HRCT presented with a mass rounded by halo sign at the right lower lung at first(8-A,white arrow), and the CT-guided transthoracic lung biopsy of the focal zone showed destructed alveolar infiltrated with granulomas and inflammatory cells, Periodic acid-Schiff (PAS) stain(+), methenamine silver stain (+), AFB(-), (8-B, black arrows, PAS stain showed the red-stained capsules of the cryptococcus, 400x magnification). After 4 weeks of fluconazole therapy, the follow-up low dose CT showed reversed halo sign in the same location as the previous lesion, which resulted from the central part absorption that made the residual lesion a ring shape (8-C).

**Fig 9:**
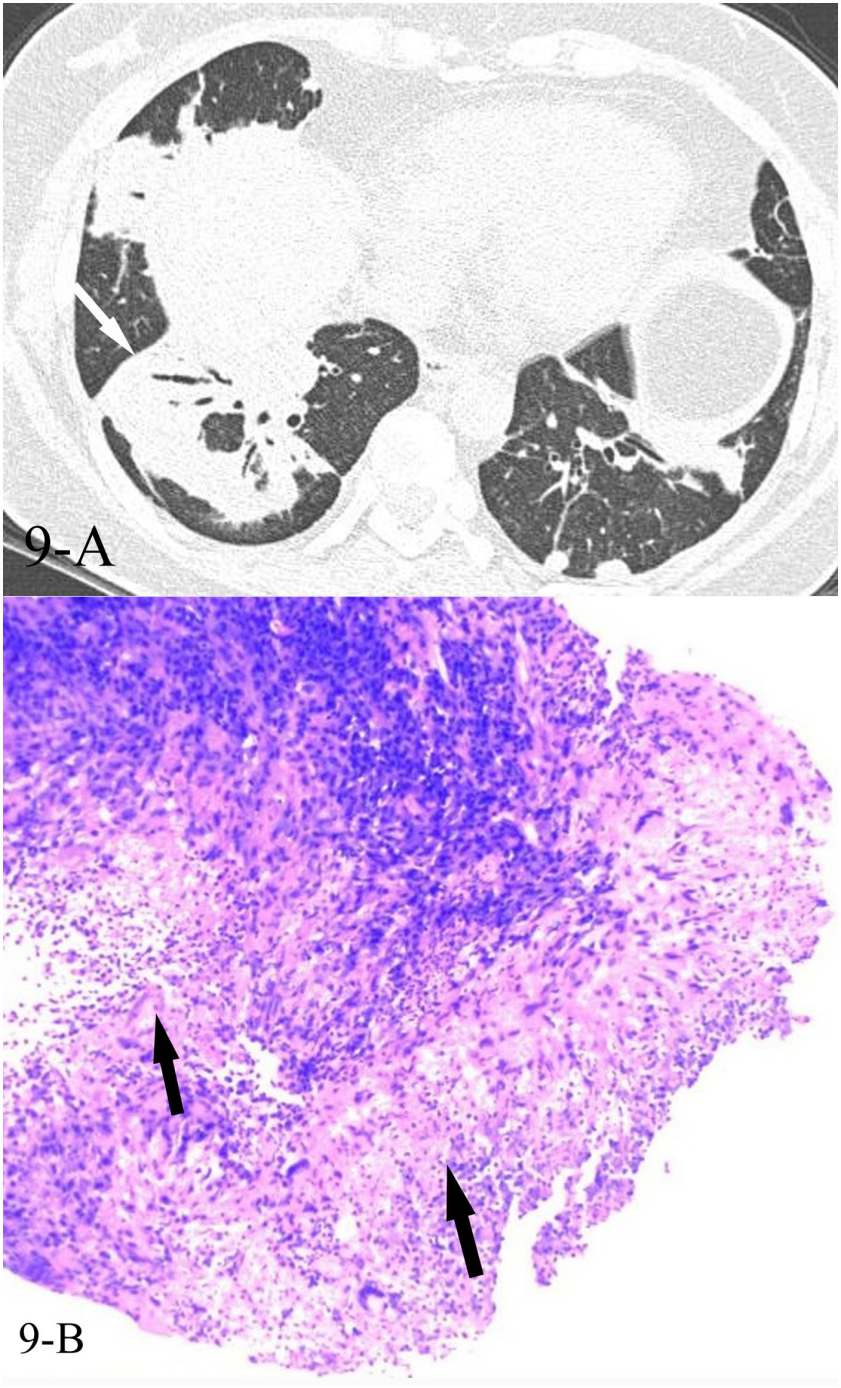
A, B. A 45-year-old female diagnosed with GPA pathologically. The HRCT showed reversed halo sign(9-A,white arrow), and the CT-guided transthoracic lung biopsy of the focal “ring” showed necrotizing granulomatous inflammation(9-B,black arrows, HE stain, 200x magnification).

In the 40 cases of pulmonary tuberculosis with reversed halo sign, 23 were diagnosed by the CT-guided transthoracic lung biopsy and the “ring” of the reversed halo sign corresponded to granulomata(Figs 1-A, 1-B, 2-B, 2-B, 3-A, 3-B, 4-A and 4-B), with or without acid-fast stain positivity, and some of them were with caseating necrosis(Figs 1-B, 2-B and 4-B). In the 7 cases of sarcoidosis with reversed halo sign, 6 were diagnosed by bronchoscopic biopsy(transbronchial lung biopsy and mucosa biopsy, Figs 5-A, 5-B, 6-A and 6-B), and 1 was by CT-guided transthoracic lung biopsy in which multiple granulomata without caseating necrosis was found in the “ring”(Fig 7-A and 7-B). In the case of pulmonary cryptococcosis, the initial HRCT demonstrated a mass surrounded by a halo sign in the right lower lung, and the CT-guided transthoracic lung biopsy of the focal zone showed destructed alveolar infiltrated with granulomas and inflammatory cells, Periodic acid-Schiff (PAS) stain(+), methenamine silver stain (+), Acid-fast Bacillus(-), and the PAS stain showed the red-stained capsules of the Cryptococcus, which confirmed the diagnosis(Fig 8-A and 8-B). However, after 4 weeks of fluconazole therapy, the follow-up low dose CT showed reversed halo sign in the same location as the previous lesion, which resulted from the absorption of the central part leaving a residual ring shaped lesion. (Fig 8-C). In the case of granulomatosis with polyangiitis(GPA) with reversed halo sign, the patient complained about earache and had high fever, CT-guided transthoracic lung biopsy of the “ring” showed necrotizing granulomatous inflammation(Fig 9-A and 9-B), and the biopsy of the left eardrum showed vasculitis.

**Fig 10:**
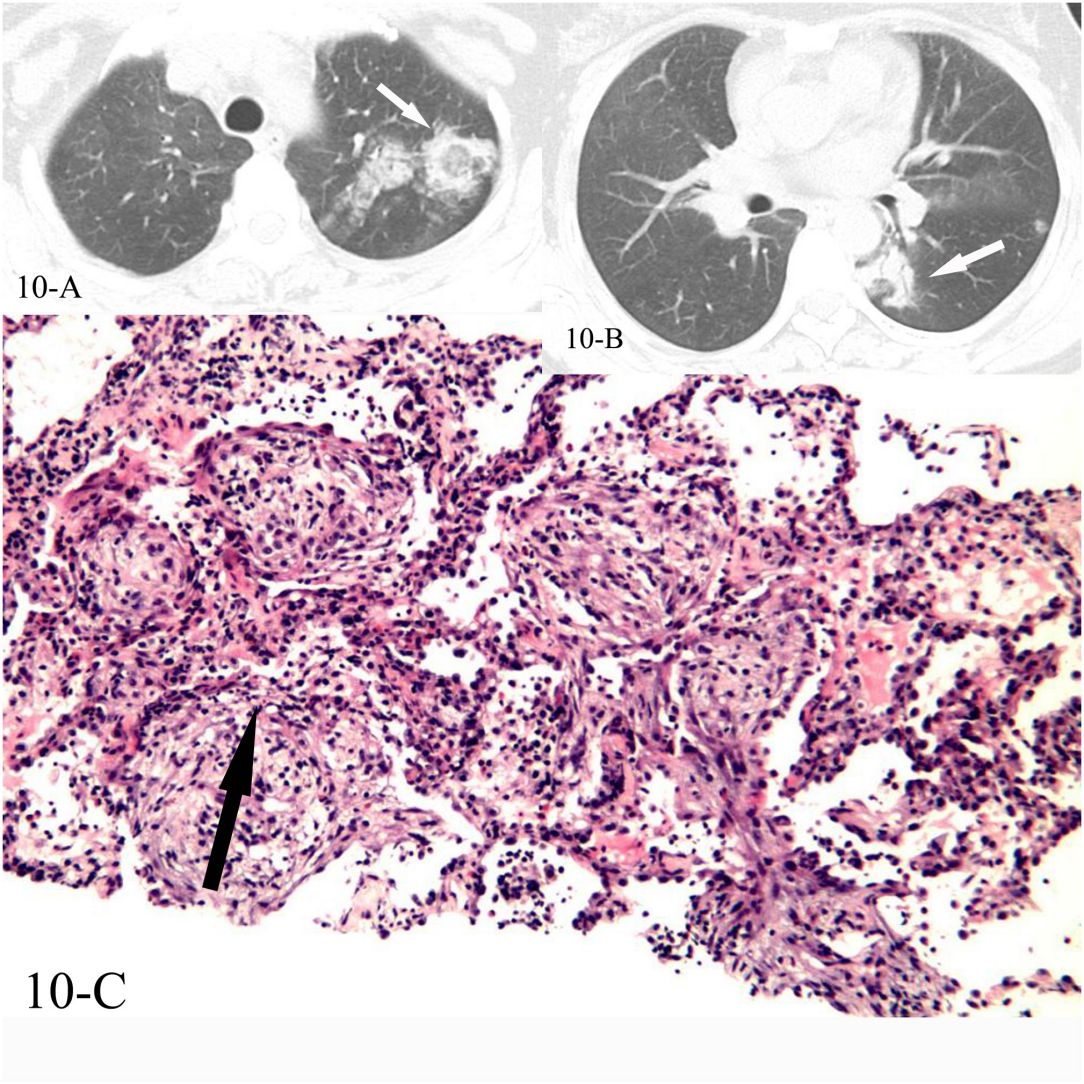
A, B, C. A 44-year-old female diagnosed with COP pathologically. The HRCT showed reversed halo sign of the left upper lobe(10-A) and the left lower lobe(10-B), and the CT-guided transthoracic lung biopsy of the “ring” of reversed halo sign in the left lower lobe showed intraluminal organizing fibrosis in distal air spaces(10-C,black arrow, HE stain, 200x magnification).

**Fig 11:**
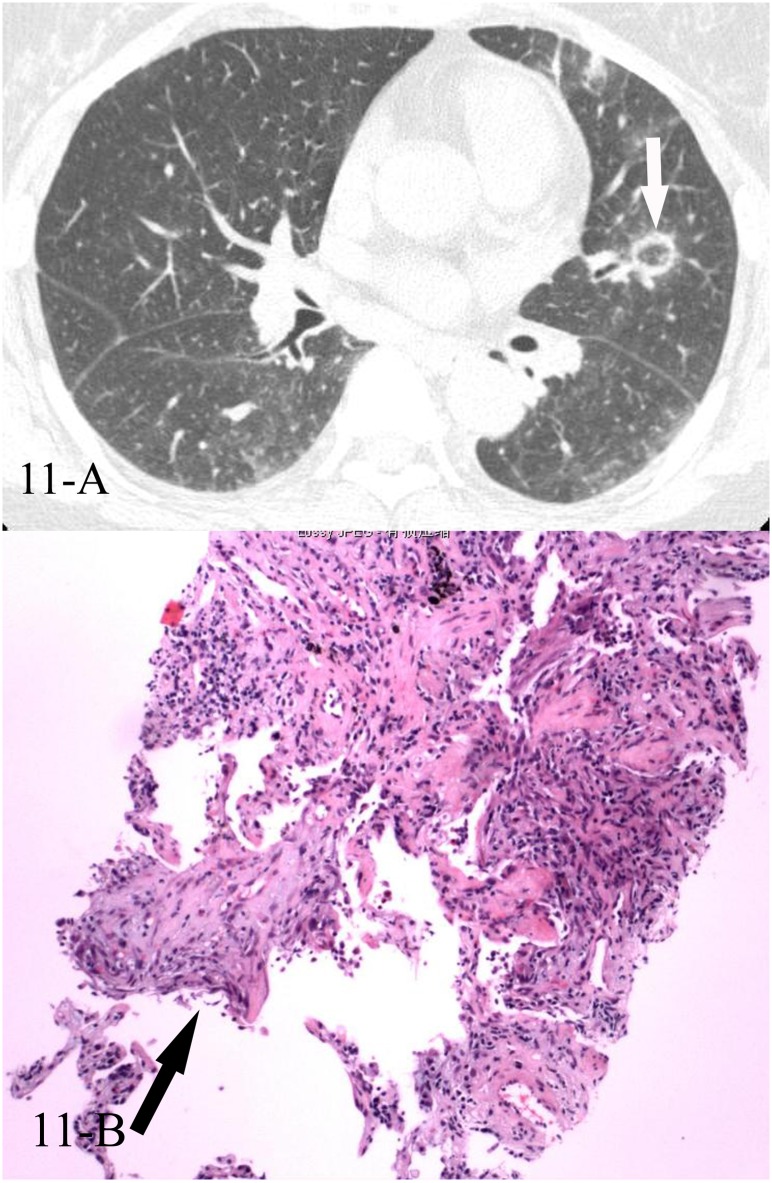
A, B. A 50-year-old female diagnosed with COP pathologically. The HRCT showed reversed halo sign of the left upper lobe(11-A), and the CT-guided transthoracic lung biopsy of the focal “ring” showed intraluminal organizing fibrosis in distal air spaces(11-B, black arrow, HE stain, 200x magnification).

**Fig 12:**
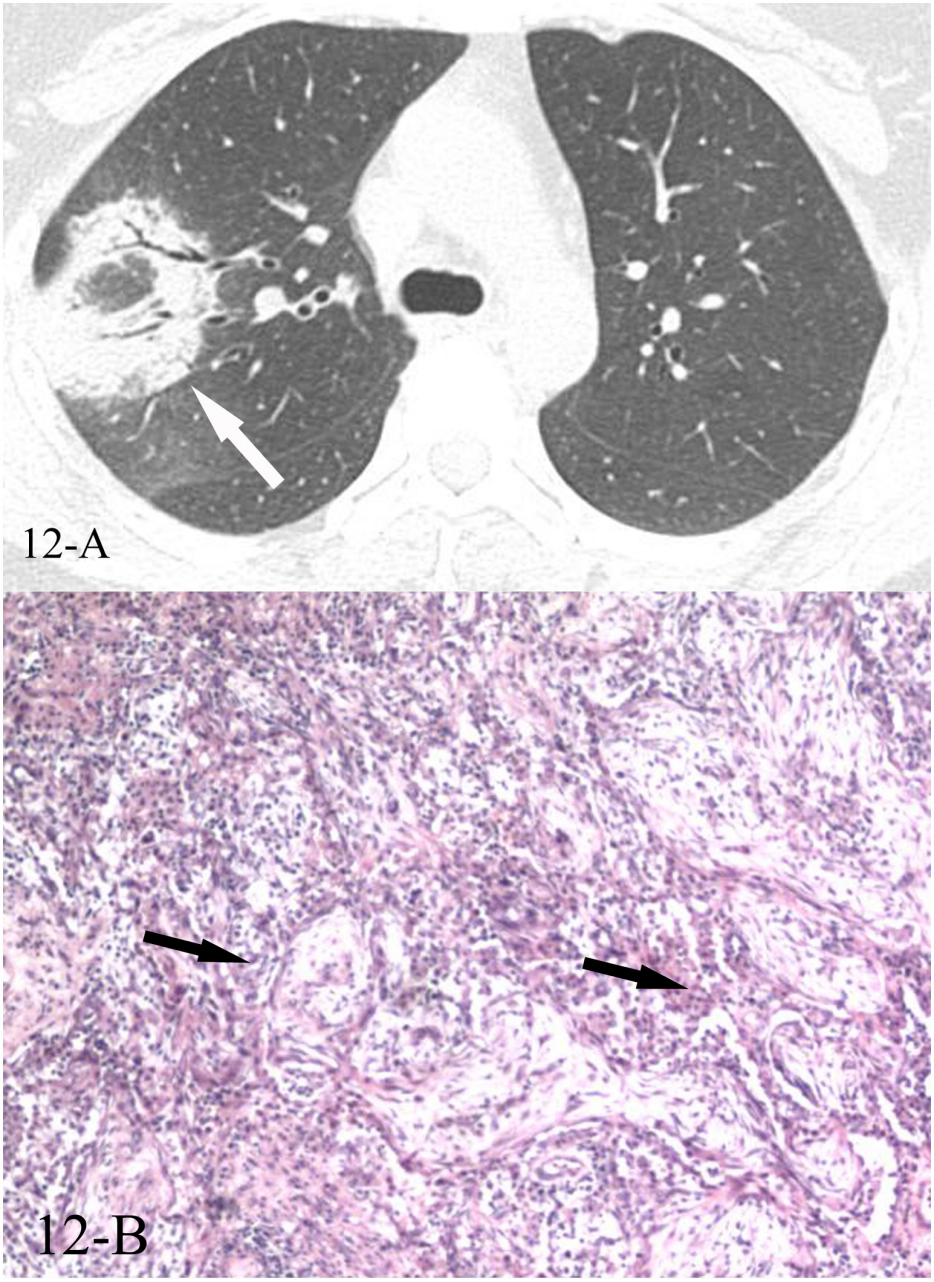
A, B. A 49-year-old female diagnosed with COP pathologically. The HRCT showed reversed halo sign of the right upper lobe(12-A), and the CT-guided transthoracic lung biopsy of the focal “ring” showed intraluminal organizing fibrosis in distal air spaces(12-B, black arrows, HE stain, 200x magnification).

**Fig 13:**
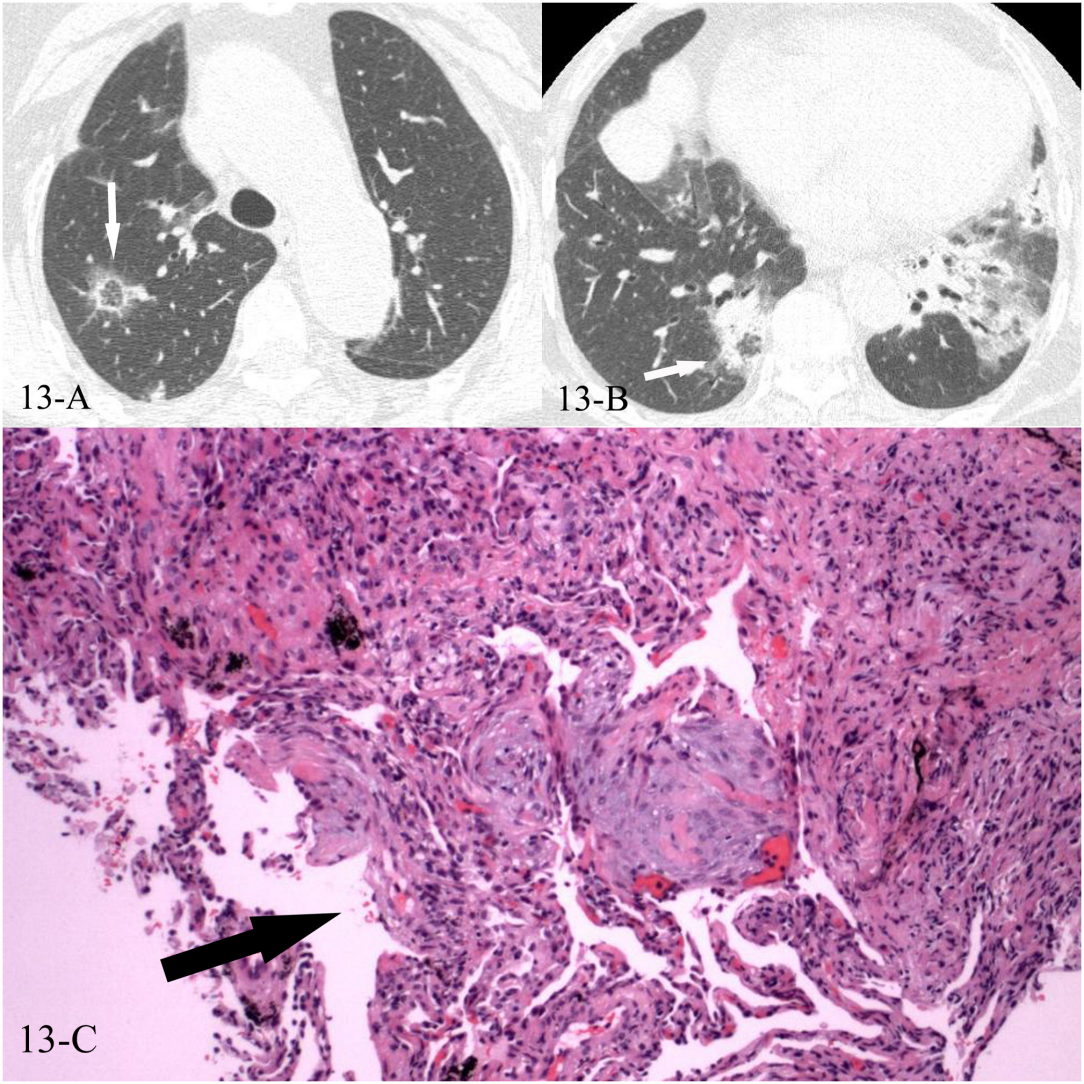
A, B, C. A 73-year-old female diagnosed with COP pathologically. The HRCT showed reversed halo sign of the right upper lobe(13-A) and the right lower lobe(13-B), and the CT-guided transthoracic lung biopsy of the focal “ring” of reversed halo sign in the right lower lobe showed intraluminal organizing fibrosis in distal air spaces(13-C, black arrow, HE stain, 200x magnification).

**Fig 14:**
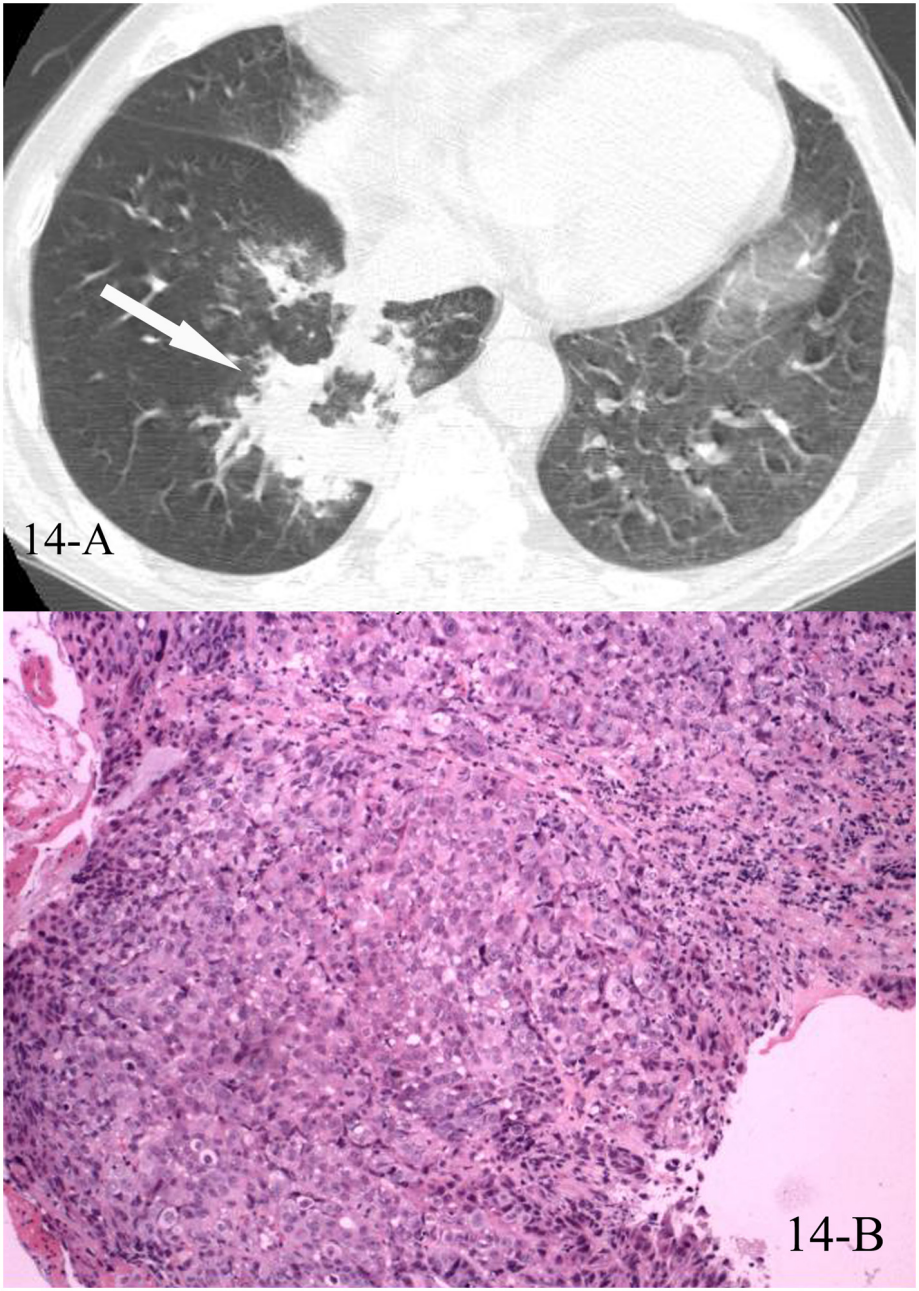
A, B. A 65-year-old male diagnosed with low differentiated adenocarcinoma pathologically. The HRCT showed reversed halo sign of the right lower lobe(14-A), and the CT-guided transthoracic lung biopsy of the focal “ring” showed nested tumor tissue with necrosis, and no normal alveolar structure was found (14-B, HE stain, 100x magnification).

**Fig 15:**
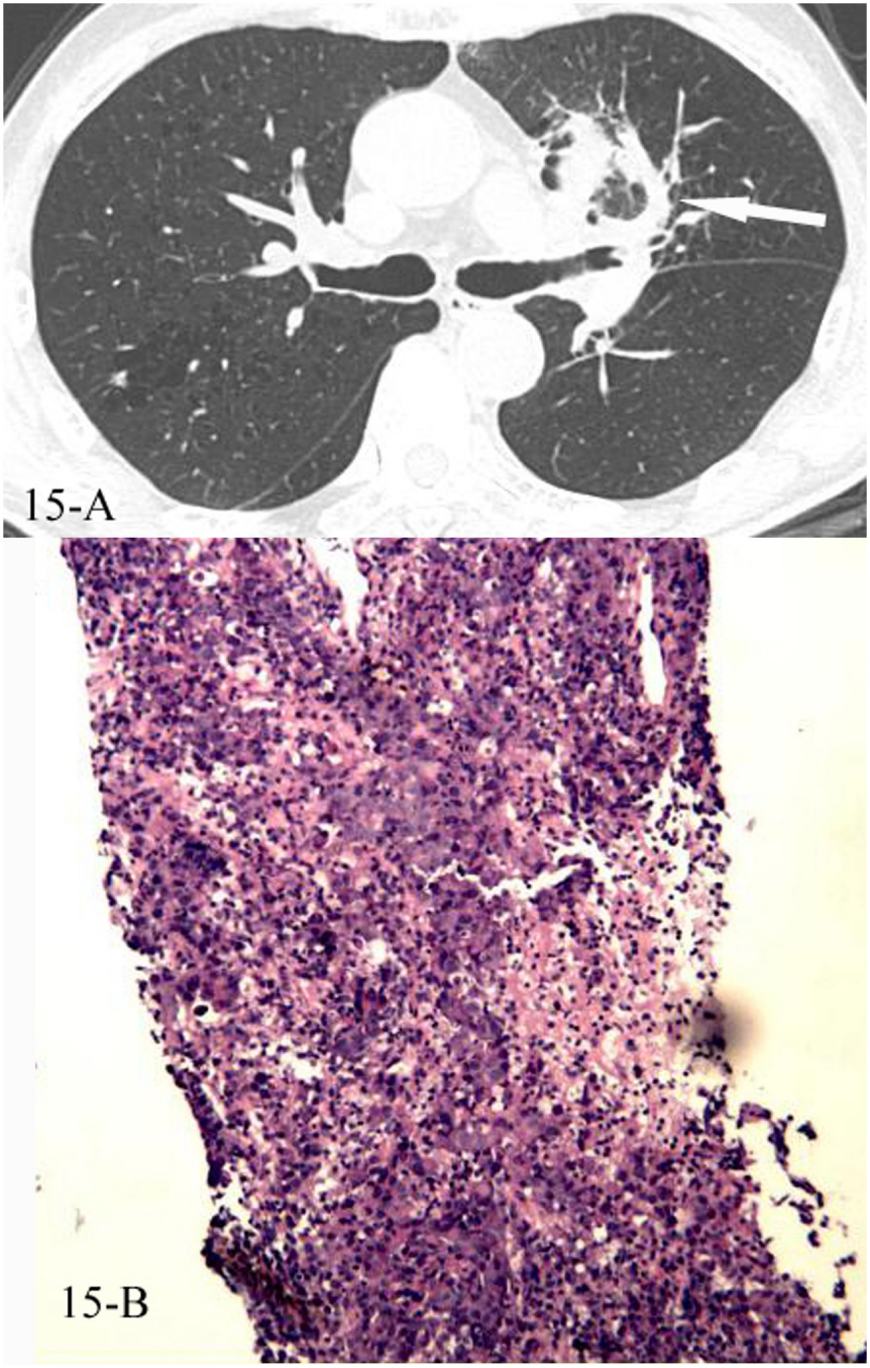
A, B. A 75-year-old male diagnosed with low differentiated adenocarcinoma pathologically. The HRCT showed reversed halo sign of the left upper lobe(15-A), and the CT-guided transthoracic lung biopsy of the focal “ring” showed diffused infiltration of tumor cells with necrosis, and no normal alveolar structure was found (15-B, HE stain, 100x magnification). The immunohistochemical staining: TTF-1(+), ALK(+), CK7(+), Napsin(+), CK5(+).

**Fig 16:**
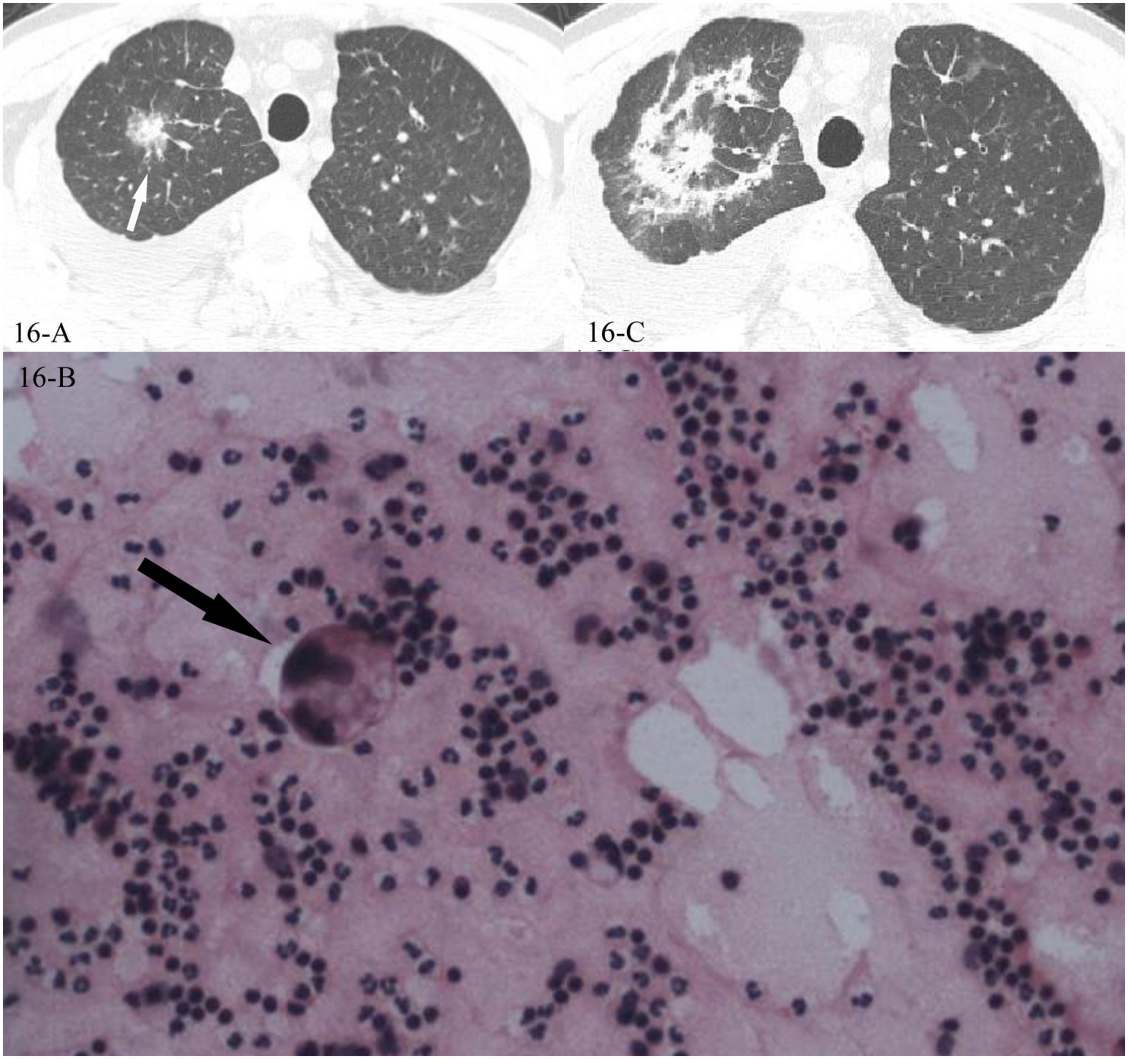
A, B, C. A 42-year-old male diagnosed with adenocarcinoma by pleural effusion, the HRCT showed a mass in the right upper lobe(16-A) and adenocarcinoma cells were found in the pleural effusion smear with the background the lymphocytes(16-B, black arrow, 200x magnification), no chemotherapy but just pleural fluid drainage was performed, and 6 weeks later the follow-up CT scan showed reversed halo sign(16-C).

In the 43 cases of cryptogenic organizing pneumonia (COP) with reversed halo sign, all were diagnosed pathologically, 41 by the CT-guided transthoracic lung biopsy and 2 by lobectomy, the “ring” of the reversed halo sign corresponded pathologically to intraluminal organizing fibrosis in distal air spaces (Figs 10-A, 10-B, 10-C, 11-A, 11-B, 12-A, 12-B, 13-A, 13-B and 13-C). In the 16 cases of lung cancer with reversed halo sign, the “ring” corresponded to nested tumor tissue with necrosis, and no normal alveolar structure was found (Figs 14-A, 14-B, 15-A and 15-B). A reversed halo sign of 1 case in the cancer group did not present at the time that the diagnosis of adenocarcinoma was initially confirmed by pleural effusion, but 6 weeks later on the follow-up CT scan, without any chemotherapy but just pleural fluid drainage during the period (Fig 16-A, 16-B and 16-C).

## Discussion

Reversed halo sign is attracting attention as a special sign on HRCT, however, due to its rareness, most of the papers were case reports and there was no systemic study based on the pathological correspondence. Reversed halo sign is a relatively nonspecific sign which can be found in different diseases, and in different phases of diseases, either the improvement of cryptococcosis (Fig 8-C) or the progression of lung cancer(Fig 16-C). However, the data showed it had higher incidence in the granulomatosis diseases than non-granulomatosis diseases, which may help with the differential diagnosis. Both pulmonary tuberculosis and invasive pulmonary fungal infection are infectious granulomatosis diseases that can present with reversed halo sign. In 2012 Marchiori et al reviewed 79 cases presented with reversed halo sign from nine tertiary hospitals of Brazil, the United States, and Canada [12], and it was the only research about the reversed halo sign in different pulmonary diseases with a large sample besides our study, 28 cases of which were of fungal infection (paracoccidioidomycosis, zygomycosis, invasive pulmonary aspergillosis, histoplasmosis, cryptococcosis) and 12 cases were pulmonary tuberculosis. Owing to the different epidemiology, our research showed a different spectrum, which contained 40 cases of tuberculosis in 108 cases presented with reversed halo sign, and only 1 case of pulmonary cryptococcosis, and the local epidemiology should be considered when making a differential diagnosis.

Reversed halo sign had different histopathological correlates in different diseases: in tuberculosis the ring corresponded to granulomata, with or without acid-fast stain positivity, and with or without caseating necrosis [11]. In sarcoidosis the ring-shaped consolidation histopathologically related to multiple granulomas [8]. In cryptogenic organizing pneumonia (COP), the central ground glass opacity corresponds to the alveolar septal inflammation and alveolar cellular desquamation with a small amount of granulation tissue in the terminal air space, and the ring-shaped consolidation corresponds histopathologically to the areas of intraluminal organizing fibrosis in distal air spaces including bronchioles, alveolar ducts, and alveolar spaces [2]. These were all consistent with our study. In pulmonary lymphomatoid granulomatosis, the central area of ground glass consists of alveolar septal inflammatory infiltrates with macrophages, lymphocytes, plasma cells and giant cells, and the ring-shape areas of consolidation corresponds to dense and homogeneous intra-alveolar inflammatory infiltrates[3]. In pulmonary zygomycosis the reversed halo sign relates to lung infarction, with a greater amount of hemorrhage at the periphery consolidation than in the center of the ground glass opacity [9]. Plus, we found pathological correspondence of the ring in different diseases that was never reported before, such as the cases of lung cancer (Figs 14-A and 15-A), and the case of cryptococcosis (Fig 8-A and 8-B). The pathological correspondence helped us better understand the CT imaging. Marchiori’s study concluded that the presence of nodular walls or nodules inside the halo of reversed halo sign was highly suggestive of granulomatous diseases, after he compared the “ring” of reversed halo sign in cryptogenic organizing pneumonia (COP) with that of the granulomatous diseases such as tuberculosis and sarcoidosis, which showed that the latter was not so smooth as the former, as the “ring” of reversed halo sign in tuberculosis and sarcoidosis were nodular walls [12]. Most of the reversed halo sign in granulomatous diseases of our study (48 out of all the 49 cases) were with rings of nodular walls; but interestingly, different from his study, in which all the sarcoidosis presented with nodular walls, our study showed a case of sarcoidosis with a smooth “ring” of the reversed halo sign(Fig 5-A). All the 43 cases of COP were with more smooth rings.

Our research has several limitations. It was a retrospective single-center study. Pathology results were not available for all the cases where a reversed halo sign was present on HRCT. However, it is the largest reported series of reversed halo sign presented in different disease, in which we observed that reversed halo sign had higher incidence in granulomatous disease compared with non granulomatous diseases, and reversed halo sign is most commonly found in pulmonary tuberculosis among the granulomatous diseases, and in cryptogenic organizing pneumonia (COP) among the non-granulomatous diseases, which might help with differential diagnosis.
